# Machine learning-based linking of bacterial genomes to optimal growth pH: a foundation for rational microbial engineering

**DOI:** 10.1186/s40104-026-01434-7

**Published:** 2026-06-11

**Authors:** Huilong Chen, Xin Yang, Muhsin AI Anas, Shuyan Feng, Yanli Lin, Gang Xu, Kuikui Ni, Fuyu Yang, Xuekai Wang

**Affiliations:** 1 College of Grassland Science and Technology, China Agricultural University, Beijing, 100193 China; 2 College of Animal Science and Veterinary Medicine, Qinghai University, Xining, 810016 China; 3 Department of Animal Nutrition and Feed Science, Faculty of Animal Science, Universitas Gadjah Mada, Yogyakarta, 55281 Indonesia; 4 College of Life Sciences, North China University of Science and Technology, Tangshan, 063210 China; 5 College of Animal Science, Guizhou University, Guiyang, 550025 China

**Keywords:** Bacterial optimal growth pH, Genomic-phenotypic linkage, Machine learning, Microbial engineering, Microbial pH adaptation

## Abstract

**Background:**

Bacterial optimal growth pH is pivotal for enzymatic activity, niche adaptation, and synthetic biology applications (e.g., probiotic design, silage fermentation). Traditional experiments are inefficient, resource-intensive, and miss most unculturable taxa, while direct genome-based prediction of this trait remains unavailable—creating a critical genomic-phenotypic gap that hinders microbial engineering.

**Results:**

We developed BactoGenopH (http://silagedb.com/BactoGenopH/), a web platform for predicting the optimal growth pH of bacteria. We curated a high-quality dataset of 3,476 samples, integrating directly measured pH values from the BacDive database and peer-reviewed literature with corresponding representative genomes from GTDB. Genomic features were extracted via Prodigal for gene prediction and HMMER for Pfam-based functional annotation, with high-importance genes retained and encoded as a binary presence/absence matrix. The XGBoost regression model exhibited robust performance: test set MAE = 0.477, RMSE = 0.666, and 88.82% accuracy (1-pH-unit tolerance); the independent validation set yielded MAE = 0.492, RMSE = 0.694, and 89.37% accuracy. SHAP analysis identified key pH-adaptation genes (e.g., Na_Ala_symp, MgtE) with well-documented roles in ion transport and pH homeostasis. The freely accessible platform supports real-time predictions via FASTA sequence input or file upload, complemented by data visualization and curated dataset browsing.

**Conclusion:**

BactoGenopH fills the unmet need for direct, phenotype-grounded bacterial optimal growth pH prediction, bridging genomic-phenotypic gaps with robust performance. This free resource accelerates trait-driven microbial research and supports rational microbial engineering.

**Supplementary Information:**

The online version contains supplementary material available at 10.1186/s40104-026-01434-7.

## Background

Bacterial optimal growth pH is a fundamental phenotypic trait that governs enzymatic activity, metabolic flux, and environmental niche occupancy [1, 2]. A robust ability to maintain intracellular pH homeostasis is essential for survival across fluctuating pH conditions and relies on a suite of evolutionarily conserved mechanisms, including proton pumps, cation/proton antiporters, amino acid decarboxylation systems, and chaperone-mediated protein repair. These core processes are encoded by defined functional genes and protein families that act as molecular fingerprints within bacterial genomes, directly governing pH adaptation, proton extrusion, proton consumption, membrane remodeling, and damage repair in response to acid or base stress [3–10]. The conservation of these pH-adaptive mechanisms and associated genes provides an innate biological basis for directly linking genomic characteristics to bacterial growth phenotypes.

This trait directly impacts the functionality of microbes in critical contexts: for example, microbial communities for synthetic biology translation require pH adaptation to maintain metabolic efficiency in industrial bioreactors [11]; silage fermentation relies on acidophilic lactic acid bacteria (LAB) to lower pH and preserve nutrient quality [12]; and probiotics must tolerate the acidic gastric environment and alkaline intestinal milieu to exert beneficial effects on livestock health [13, 14]. Despite its importance, traditional experimental methods for determining optimal growth pH are time-consuming, costly, and restricted to culturable taxa, leaving the vast majority of uncultured microbes without characterized phenotypic information [15–17]. Meanwhile, genomic data continue to accumulate rapidly, with more than 100,000 bacterial genomes now available in GTDB [18].

Previous studies have explored machine learning (ML)-based approaches to predict bacterial pH preferences from genomic features. For example, Ramoneda et al. [19] developed an XGBoost-based model [20] using presence–absence patterns of Pfam-annotated gene families and indirectly inferred pH values derived from environmental abundance peaks to predict bacterial pH ranges. While these investigations confirmed the feasibility of genome–phenotype linkage, two key constraints remain: (1) reliance on indirect pH proxies rather than directly measured values of optimal growth pH; and (2) use of predefined gene sets that may limit applicability across diverse bacterial lineages. We therefore established a predictive framework using well-validated, curated phenotypes and comprehensive functional annotations to support reliable optimal growth pH prediction for both cultured and uncultured bacteria.

Synthetic biology has emerged as a powerful interdisciplinary field that applies engineering principles to design, construct, and optimize biological systems [21]. It has already demonstrated transformative potential: next-generation probiotics engineered via synthetic biology to enhance acid tolerance and targeted nutrient synthesis, rationally designed microbial consortia for optimized fermentation processes, and cell-free systems (e.g., microbial metabolites, membrane vesicles) enabling antibiotic-free disease control [22–24]. A key bottleneck in advancing these applications is the lack of high-throughput tools to predict phenotypic traits (e.g., pH adaptation) from genomic data—critical for prioritizing candidate microbes or engineering targets. By integrating ML-driven prediction with functional gene identification, our platform fills this gap, providing a resource to accelerate synthetic biology research.

The primary objectives of this work were: (1) to curate a large, high-quality dataset of bacterial optimal growth pH values paired with corresponding genomes; (2) to develop an ML model with high predictive accuracy and generalizability; (3) to identify key genes mediating pH adaptation via interpretability analysis (SHAP); and (4) to build a user-friendly web platform that enables real-time prediction and supports synthetic biology applications. Here, we present BactoGenopH, a comprehensive resource that bridges genomic-phenotypic gaps and provides a foundation for rational microbial engineering.

## Methods

### Dataset curation

The measured optimal growth pH values were collected from two types of sources. First, optimal growth pH data were retrieved from the BacDive database (https://bacdive.dsmz.de/, downloaded on November 28, 2023) [25], yielding an initial set of 5,621 records. To further expand the dataset, we performed literature retrieval using Google Scholar and Web of Science with keywords including “Bacteria*”, “Growth”, “Optim**”, and “pH”, combined with regular expression‐based screening. We focused on data descriptor papers and review articles, which typically provide large‐scale curated phenotypic data. In total, two publications were identified as suitable for data supplementation: (1) a data resource paper published in *Scientific Data*, from which we retrieved 4,255 curated records of optimal growth pH via the public data repository associated with the study [26]; and (2) a review article that provided seven optimal growth pH records for acidophilic bacteria [10].

A stringent data curation pipeline was then applied to process the raw dataset, including outlier handling, missing value handling, harmonization processing, and removal of duplicate entries. For outlier handling, we corrected typographical errors (e.g., “4.4.” to 4.4) and removed biologically invalid records with negative pH values. For missing value handling, entries with missing pH values (NA) were excluded to ensure data integrity. For harmonization processing, data reported as a range (e.g., 3.0–4.0) were represented by their median value [19, 26], and all pH values were standardized to two decimal places. For removal of duplicate entries, we retained only one entry per species with priority given to type strains, isolates with a single measured pH value (rather than ranges), strains with high-quality complete genomes, and records representative of the most strains; after merging BacDive and literature data, duplicates were removed based on species name and optimal pH value, and any species with conflicting pH values across sources were discarded entirely.

**Fig. 1:**
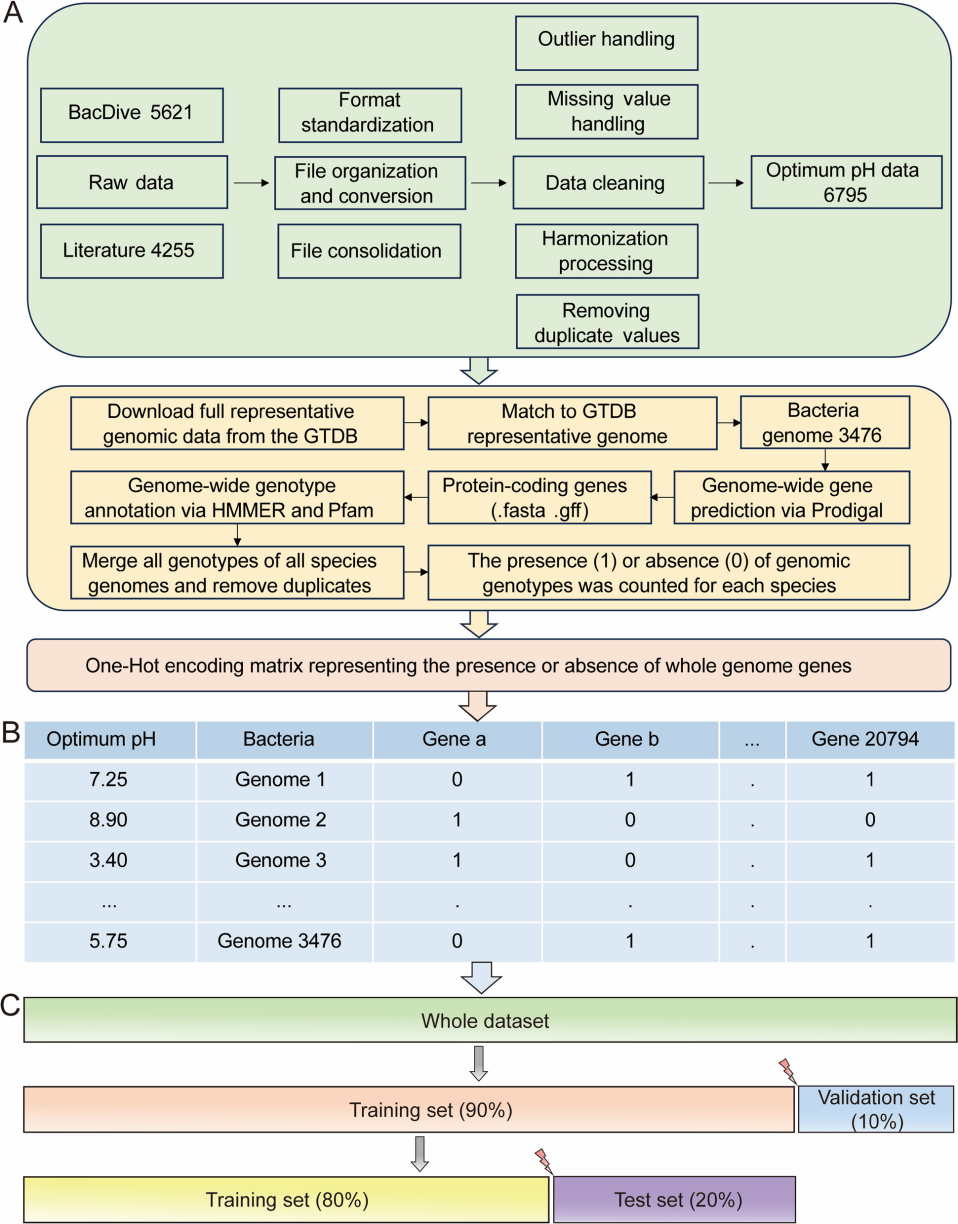
Data integration and preprocessing workflow for pH-genome machine learning model training. **A** Schematic diagram of the workflow for processing and integrating measured optimal growth pH values with GTDB representative genomic data to form a machine learning training dataset. **B** Schematic of the large matrix from matching genomic and optimal pH data. **C** Schematic diagram of secondary splitting of one-hot encoding matrix data

Representative bacterial genomes were downloaded directly from the Genome Taxonomy Database (GTDB release 214) at https://data.gtdb.ecogenomic.org/releases/release214/214.1/genomic_files_reps/ using the file gtdb_genomes_reps_r214.tar.gz, which contains pre‐processed, high‐quality, non‐redundant representative genomes derived from diverse sources including isolates and metagenome‐assembled genomes (MAGs) for each bacterial species [18]. Phenotype–genome pairing was achieved by exact string matching of Latin species names. After complete curation and pairing, the final dataset contained 3,476 high‐quality matched bacterial samples. Raw genomic data were preprocessed to generate a standardized input matrix for model training. First, Prodigal (version 2.6.3) [27] was used for de novo gene prediction with default parameters (-g 11, -p single), yielding all candidate genes across all genomes. HMMER (version 3.3.2) [28] was then applied for Pfam-based functional annotation with parameters (--noali -T 10), with all genes (20,794 unique genes that could be annotated via Pfam database (version 36) [29]) retained as binary features (1 = gene present, 0 = gene absent) (Fig. 1A).

This genomic feature matrix was merged with the optimal pH phenotype vector to form a 3,476 × 20,795 dataframe, where rows represent bacterial samples and columns include 20,794 genomic features plus 1 phenotypic label (optimum pH) (Fig. 1B). The final 3,476 data was split into a training set (90%) and an independent validation set (10%) using stratified random sampling to maintain the distribution of pH groups. The training set was further partitioned into a training subset (80%) and a test subset (20%) for model tuning and initial evaluation (Fig. 1C).

### Feature selection

To enable a fair comparison with the existing study, we used the XGBoost algorithm as the core predictor for model construction and analysis. To mitigate overfitting, reduce computational complexity, and avoid reliance on preselected gene sets, a feature selection procedure was performed strictly on the training set only to prevent data leakage and optimistic bias. Specifically, we first trained an initial XGBoost model using the full set of 20,794 genes (represented as a binary presence/absence matrix) on the training partition alone, then computed gene importance scores using the .feature_importances_ function [20]. Genes with importance scores above the mean were retained as the final feature set. All selected features were represented in a binary format (1 = present, 0 = absent). The test and independent validation sets remained completely unseen throughout the feature prioritization step and were only used for final model evaluation.

### Model development

To ensure consistency with the feature selection workflow, XGBoost (version 2.0.3) was used for regression modeling of bacterial optimal growth pH. Hyperparameter optimization was implemented via scikit-optimize with Bayesian optimization [30]. The hyperparameter search space encompassed: n_estimators (50–1000), learning_rate (0.01–0.2, log-uniform prior), max_depth (3–15), min_child_weight (1–10), gamma (0.01–5, log-uniform prior), subsample (0.5–1.0), colsample_bytree (0.5–1.0), reg_alpha (0.01–1, log-uniform prior), and reg_lambda (1–5, log-uniform prior). A tenfold cross-validation was conducted on the training subset, where the objective function minimized the mean squared error (MSE), calculated as:

1 $$MSE=\frac{1}{n}\sum\limits_{i=1}^{n}{({y}_{i}-{\widehat{y}}_{i})}^{2}$$

where $n$ is the number of samples, ${y}_{i}$ is the measured optimal growth pH, and ${\widehat{y}}_{i}$ is the model-predicted optimal growth pH.

Cross-validation was performed via negative MSE scoring, and the optimal hyperparameter combination was selected based on the lowest cross-validated MSE, and these parameters were used to construct the final XGBoost model.

### Model evaluation

The performance of the preconstructed XGBoost model was assessed on the test set using a panel of regression metrics, including MSE, mean absolute error (MAE), root mean squared error (RMSE), and the coefficient of determination (*R*^2^). These metrics were mathematically defined as follows:

2 $$MAE=\frac{1}{n}\sum\limits_{i=1}^{n}\left|{y}_{i}-{\widehat{y}}_{i}\right|$$

3 $$RMSE=\sqrt{\frac{1}{n}\sum\limits_{i=1}^{n}{({y}_{i}-{\widehat{y}}_{i})}^{2}}$$

4 $${R}^{2}=1-\frac{{\sum}_{i=1}^{n}{({y}_{i}-{\widehat{y}}_{i})}^{2}}{{\sum}_{i=1}^{n}{({y}_{i}-\overline{y})}^{2}}$$

where $n$ is the number of samples, ${y}_{i}$ is the measured optimal growth pH, ${\widehat{y}}_{i}$ is the model-predicted optimal growth pH, and $\overline{y }$ is the mean of the measured pH values.

To provide additional actionable insights, we innovatively transformed this regression task into a pseudo-classification task to derive an accuracy metric. In line with prior literature, an error tolerance of 1 pH unit was permitted for accuracy calculation [19, 31, 32], and the initial model achieved 88.82% accuracy on the test set under this tolerance threshold. Furthermore, we quantified the model’s accuracy within narrower error ranges (0.5 to 2.0 pH unit) to comprehensively characterize its predictive precision.

### Interpretability analysis

SHapley Additive exPlanations (SHAP) analysis [33] was performed to identify key genes driving pH predictions. SHAP values quantify the contribution of each gene to the model’s output, with positive values increasing predicted pH and negative values decreasing it. The top 20 genes with the highest absolute SHAP values were selected for functional enrichment analysis using Pfam database [29].

### Web platform development

The BactoGenopH web platform was developed as a publicly accessible tool for predicting bacterial optimal growth pH. The backend was constructed using the Django framework in Python, while the frontend interface was designed with HTML5, CSS3, and JavaScript to ensure cross‑device compatibility and intuitive user interaction [34, 35]. The trained XGBoost model was deployed using the pickle module for efficient loading and inference, and matplotlib was employed to generate visualizations.

**Fig. 2:**
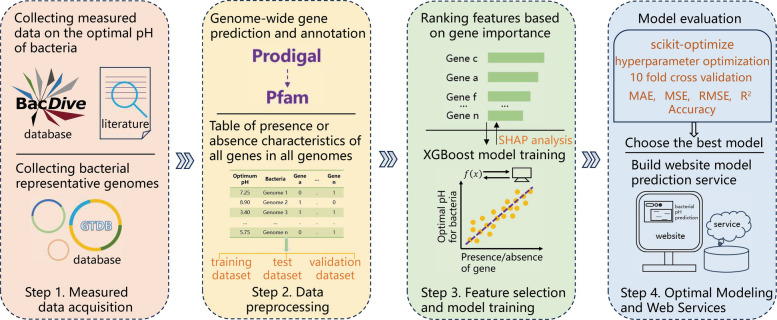
Workflow for predicting the optimal pH for bacterial growth

The platform accepts genomic sequences in FASTA format with a minimum valid length of 20,000 bp and a recommended length of at least 100,000 bp for reliable prediction, and provides real-time inference of optimal growth pH upon sequence submission. Additional functions include visualization, access to curated datasets and statistical summaries, and optional email notifications for task completion. The BactoGenopH platform is freely available at http://silagedb.com/BactoGenopH/, and the overall workflow of this study is illustrated in Fig. 2.

## Results

### Classification and distribution of bacterial optimal growth pH

**Fig. 3:**
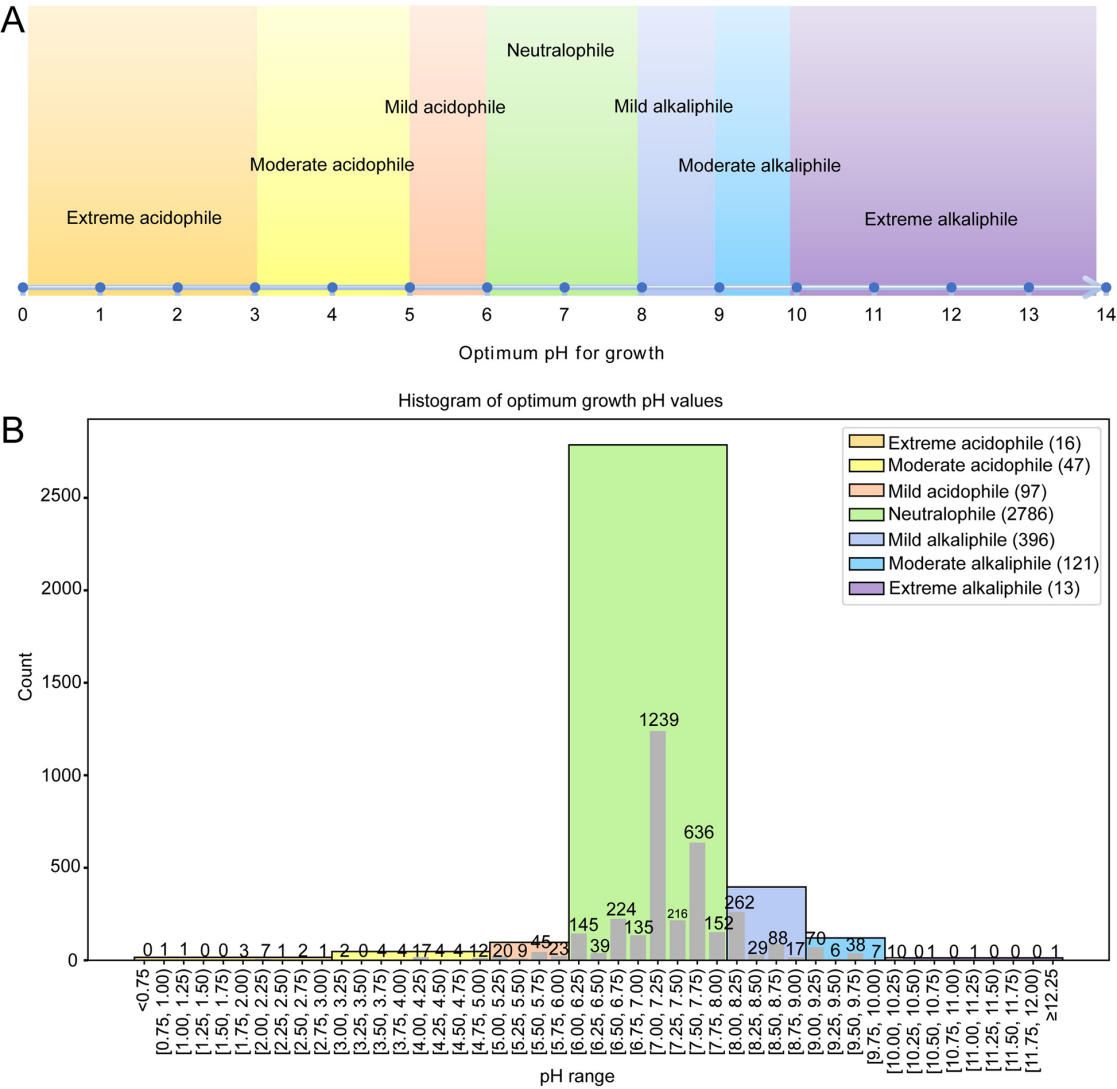
Categorization of microorganisms into types based on optimal growth pH ranges. **A** Classification of literature-reported microorganisms by optimal pH values. **B** Histogram of the count of microorganisms per category

Based on literature-defined pH ranges [36, 37], the 3,476 bacteria were categorized into seven functional groups (Fig. 3A): extreme acidophiles (pH < 3.0, *n* = 16), moderate acidophiles (pH 3.0–5.0, *n* = 47), mild acidophiles (pH 5.0–6.5, *n* = 97), neutralophiles (pH 6.0–8.0, *n* = 2,786), mild alkaliphiles (pH 8.0–9.0, *n* = 396), moderate alkaliphiles (pH 9.0–10.0, *n* = 121), and extreme alkaliphiles (pH ≥ 10.0, *n* = 13). The histogram of group counts (Fig. 3B) revealed a dominant neutralophile fraction (80.15%), consistent with the prevalence of neutral pH niches in natural and agricultural ecosystems [25, 26], while extreme pH groups were less abundant—reflecting the technical challenges of isolating and culturing extremophilic bacteria.

### Taxonomic distribution of the dataset

**Fig. 4:**
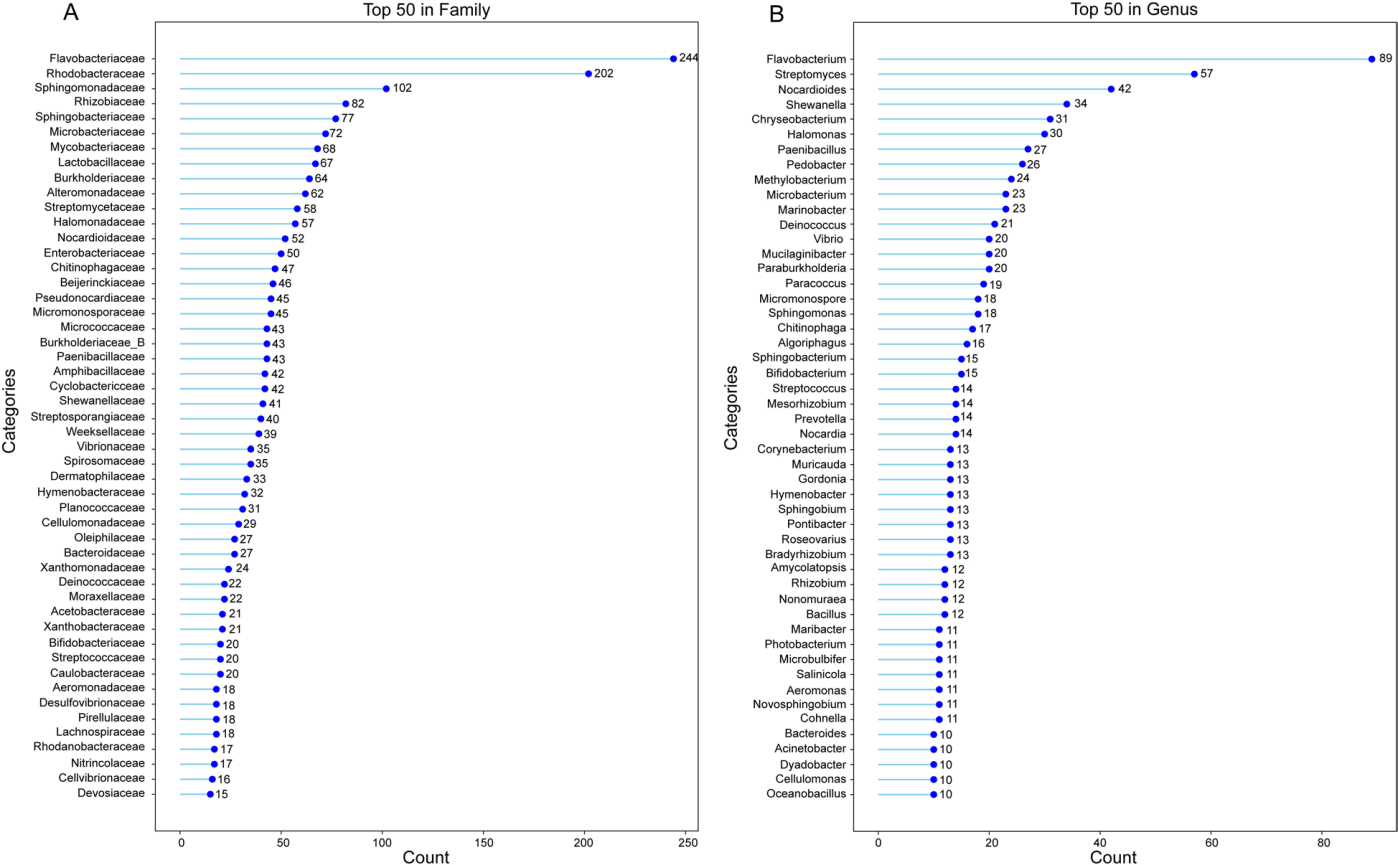
Distribution plot of the top 50 families and genera by total count of representative genomes matched to optimal growth pH

At all levels of biological classification, the dataset exhibited broad taxonomic coverage (Fig. 4; Figs. S1–S3; Tables S1–S5). The top 50 families accounted for 67% of all samples, with Flavobacteriaceae (7.02%), Rhodobacteraceae (5.81%), Sphingomonadaceae (2.93%) being the most abundant (Fig. 4A). At the genus level, *Flavobacterium* (2.56%), *Streptomyces* (1.64%), and *Nocardioides* (1.21%) were the most prevalent (Fig. 4B). Supplementary figures S1–S3 provide complete taxonomic distribution profiles at the phylum, class, and order levels, verifying the dataset’s taxonomic breadth (covering 37 phyla, 78 classes, 213 orders, 461 families, 1,492 genera).

### Phenotypic-genomic association

**Fig. 5:**
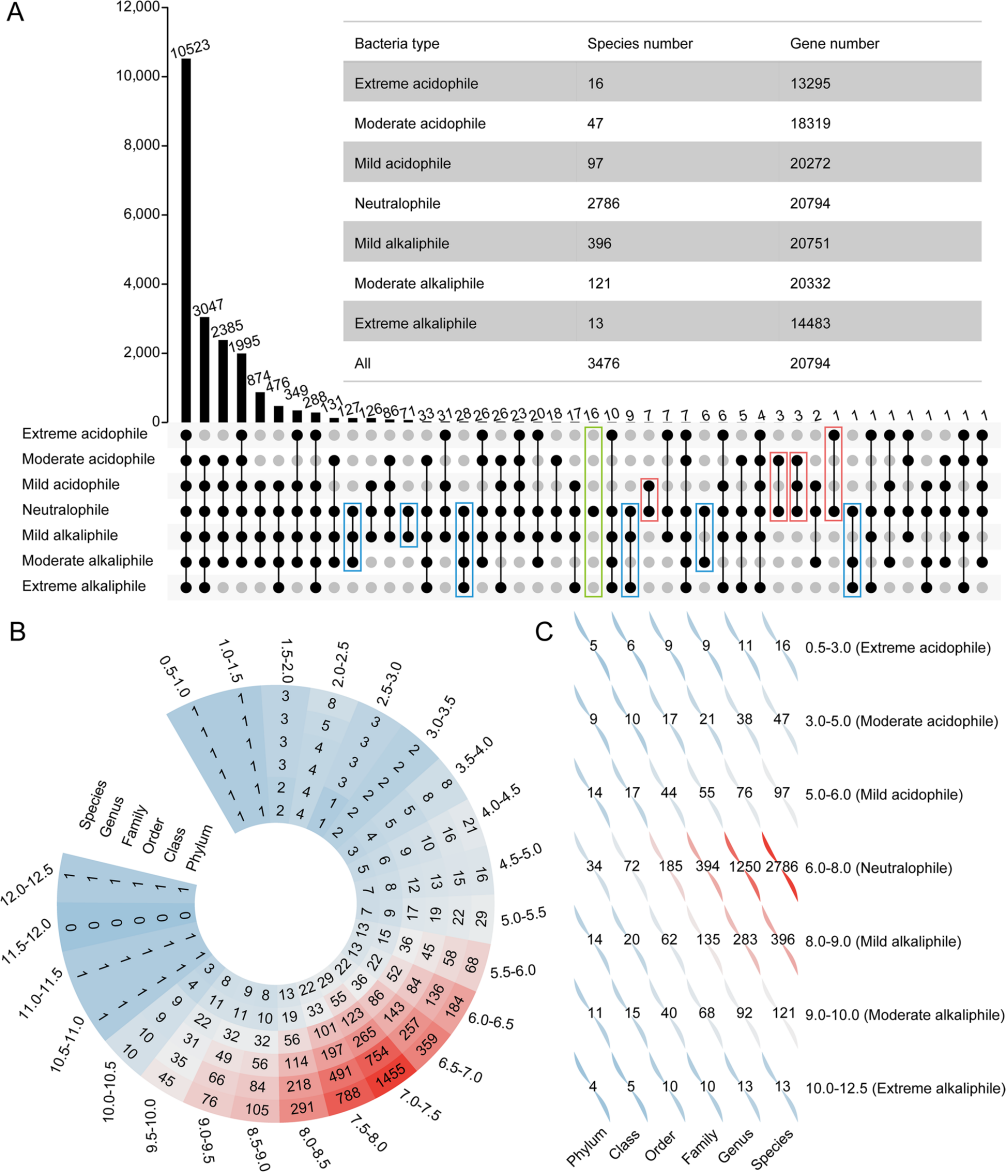
Integrated analysis of genomic and pH-related data. **A** Comparison of total genes among different types of bacteria. In the UpSet plot, the gene set highlighted by the blue box represents genes absent in acidophile genomes, the red box indicates genes absent in alkaliphile genomes, and the green box denotes genes unique to neutralophile genomes. **B** Distribution of counts of all hierarchical classification levels across optimal growth pH intervals with 0.5-unit spacing. **C** Distribution of the number of microbial taxa across different biological classification levels

UpSet plot analysis (Fig. 5A) of gene overlap across the seven pH groups identified 10,523 shared core genes (accounting for 50.61% of all 20,794 genes) that were present in all groups (Table S6). Among the non‑shared genes, 242 genes were absent exclusively in acidophile genomes (Table S7), 14 genes were absent exclusively in alkaliphile genomes (Table S8), and 16 genes were present only in neutralophile genomes (Table S9). While core genes likely sustain fundamental bacterial metabolism, the non‑shared (accessory or differential) genes are likely the primary drivers of adaptation to specific pH niches. Notably, the number of non‑shared genes may decrease as more acidophile and alkaliphile genomes become available; nevertheless, these observations remain highly meaningful and can provide valuable candidate targets for gene editing and related mechanistic studies.

To visualize phenotypic-taxonomic correlations, a circular heatmap (Fig. 5B) was generated by binning optimal pH into 0.5-unit intervals and mapping taxonomic abundance (phylum, class, order, family, and genus level) to each interval. The heatmap revealed clear niche differentiation: the various microbial groups were mainly enriched in the pH range of 6.0 to 9.0 (neutralophile and mild alkaliphile). A second heatmap (Fig. 5C) quantified the count distribution of the seven pH groups across major taxonomic, further confirming that above conclusion on pH adaptation.

### XGBoost model training

**Fig. 6:**
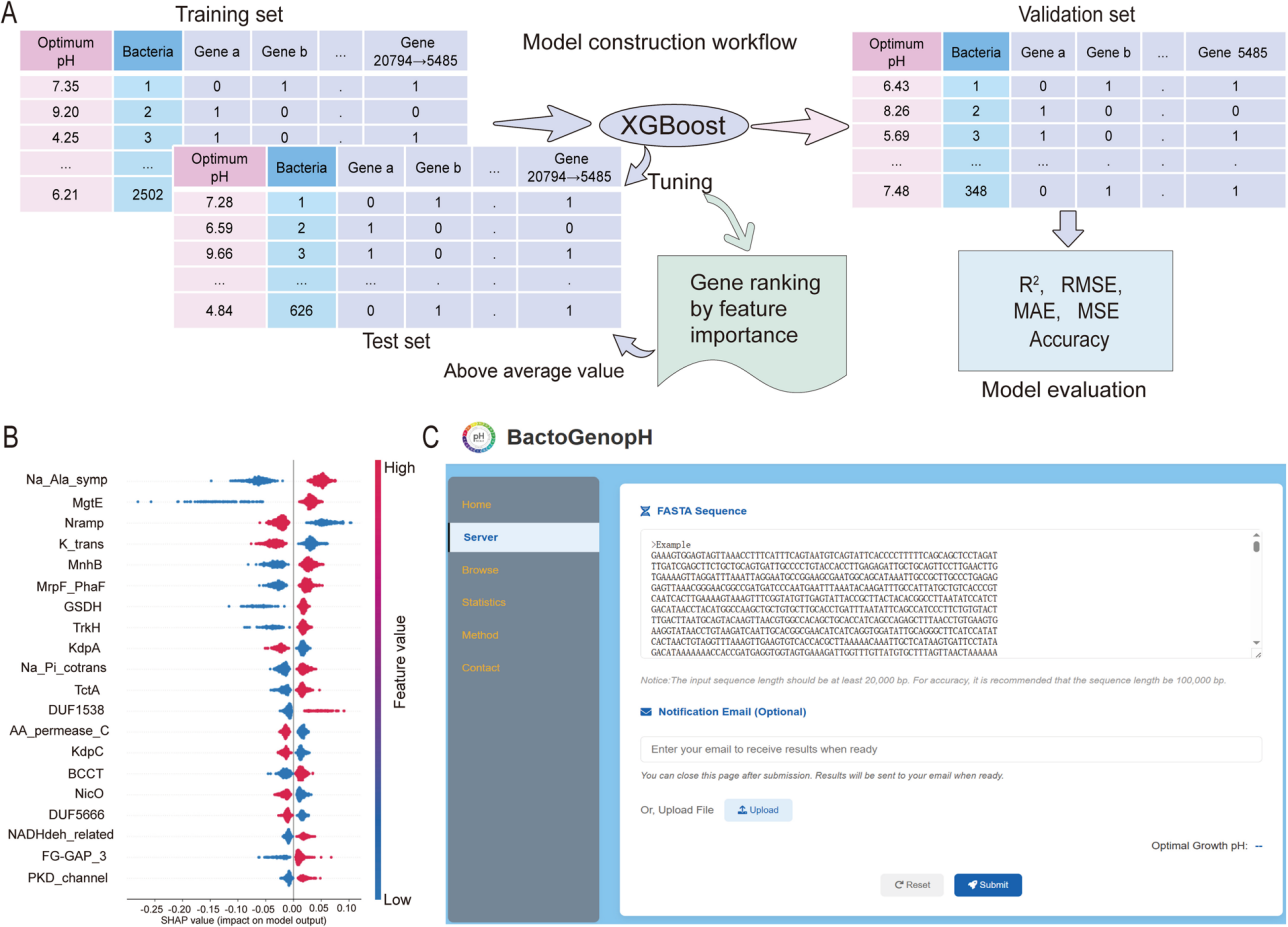
Workflow, interpretability analysis, and platform interface for the XGBoost-based bacterial optimal pH prediction model. **A** Workflow for XGBoost model construction and feature gene screening. **B** SHAP analysis of the top 20 most influential genes. **C** Server interface of the BactoGenopH platform

To avoid bias and ensure model generalizability, the dataframe was split into three subsets using stratified random sampling. The training set accounts for 72% of total samples with *n* = 2,502 and was used for initial model training and hyperparameter optimization. The test set accounts for 18% of total samples with *n* = 626 and was used for intermediate performance evaluation. The independent validation set accounts for 10% of total samples with *n* = 348 and was used for final unbiased validation of the BactoGenopH tool (Fig. 6A). Stratification was critical to maintain the representation of rare groups (e.g., extreme acidophiles/alkaliphiles) in each subset, preventing model overfitting to dominant neutralophiles. XGBoost was selected as the regression algorithm for optimal pH prediction, with a two-stage optimization workflow. Post-training, the XGBoost’s .feature_importances_ function was used to calculate the importance score of each of the 20,794 genes. Only genes with scores exceeding the mean importance value (*n* = 5,485) were retained as final features—reducing computational complexity while preserving predictive information (avoiding overfitting from redundant or low-impact genes). The total time required for the two rounds of model training (first on the 20,794 genes and then on the selected 5,485 genes) was approximately 1 d and 16 h, which was completed on a high-performance computing cluster.

### Identification of key pH-adaptation genes via SHAP analysis

SHAP was applied to interpret the optimized model and identify functionally relevant genes driving pH predictions. SHAP analysis of the top 20 genes with the greatest contribution to XGBoost model predictions revealed strong biological relevance (Fig. 6B; Table 1). Most of these genes are well-documented in mediating bacterial pH adaptation: for instance, Na_Ala_symp (sodium/alanine symporter family) facilitates alanine transport coupled to sodium ion movement, a process critical for maintaining intracellular pH balance; MgtE (transmembrane Mg^2+^ transporter) regulates cellular uptake of Mg^2+^ and other divalent cations, a key mechanism in mitigating pH-induced osmotic stress [19, 29]. Together, these observations demonstrate that the contributions of such key genes enable the model to effectively distinguish bacterial pH preferences.

**Table 1:** Pfam-based functional description of top 20 genes from SHAP analysis

Target name	Description of target	Accession
Na_Ala_symp	Sodium:alanine symporter family	PF01235.21
MgtE	Divalent cation transporter	PF01769.20
Nramp	Natural resistance-associated macrophage protein-like	PF01566.22
K_trans	K^+^ potassium transporter	PF02705.20
MnhB	Domain related to MnhB subunit of Na^+^/H^+^ antiporter	PF04039.17
MrpF_PhaF	Multiple resistance and pH regulation protein F (MrpF/PhaF)	PF04066.17
GSDH	Glucose/Sorbosone dehydrogenase	PF07995.15
TrkH	Cation transport protein	PF02386.20
KdpA	Potassium-transporting ATPase A subunit	PF03814.19
Na_Pi_cotrans	Na^+^/Pi-cotransporter	PF02690.19
TctA	Tripartite tricarboxylate transporter TctA family	PF01970.20
DUF1538	Protein of unknown function (DUF1538)	PF07556.15
AA_permease_C	C-terminus of AA_permease	PF13906.10
KdpC	K^+^ -transporting ATPase, c chain	PF02669.19
BCCT	BCCT, betaine/carnitine/choline family transporter	PF02028.21
NicO	High-affinity nickel-transport protein	PF03824.20
DUF5666	Domain of unknown function (DUF5666)	PF18914.4
NADHdeh_related	NADH dehydrogenase I, subunit N related protein	PF10125.13
FG-GAP_3	FG-GAP-like repeat	PF13517.10
PKD_channel	Polycystin cation channel	PF08016.16

### Model performance evaluation

The optimized XGBoost model (trained on 5,485 selected genes) exhibited robust performance across both test and independent validation sets (Table 2). On the test set, the model achieved a MAE of 0.477, MSE of 0.443, RMSE of 0.666, and *R*^2^ of 0.35. Performance remained consistent on the independent validation set (MAE = 0.492, MSE = 0.481, RMSE = 0.694, *R*^2^ = 0.416)—confirming the model’s generalizability and resistance to overfitting.

**Table 2:** Performance of the XGBoost model on the test set and validation set

Model evaluation index	Test set	Validation set
MAE	0.47749	0.492234
MSE	0.443375	0.481866
RMSE	0.665864	0.694166
*R*^2^	0.350277	0.416336

To provide actionable insights for applied scenarios (e.g., probiotic screening), the regression task was converted to a pseudo-classification task by defining accuracy as the proportion of predictions within a specified pH tolerance (Table 3). As expected, accuracy increased with broader tolerance: ± 0.5 pH units: 65.5% (test set) and 63.2% (validation set); ± 1.0 pH units: 88.8% (test set) and 89.4% (validation set); ± 2.0 pH units: 98.4% (test set) and 98% (validation set). Notably, even at the strict ±0.5 threshold, accuracy exceeded 65% and supporting the model’s utility for high-precision applications.

**Table 3:** Accuracy of the model evaluated at different precision thresholds

Allowable error	Accuracy on the test set	Accuracy on the validation set
0.5 pH units	0.654952	0.632184
0.6 pH units	0.728435	0.724138
0.7 pH units	0.779553	0.781609
0.8 pH units	0.837061	0.83046
0.9 pH units	0.86901	0.867816
1.0 pH units	0.888179	0.893678
2.0 pH units	0.984026	0.979885

### BactoGenopH web platform implementation

The BactoGenopH platform (http://silagedb.com/BactoGenopH/) was developed to provide open and convenient access to the bacterial optimal growth pH prediction model (Fig. 6C). The platform consists of multiple structured pages, including a Home page with detailed introductions and workflow illustrations, a Server page for core prediction services, a Browse page displaying study-related information, static figures, and a complete list of Python packages and versions used for model construction, a Statistics page presenting bioinformatics statistical charts generated in this study, a Method page describing detailed analytical procedures and relevant web resources, and a Contact page for user feedback and technical support.

On the Server page, users can perform pH prediction by submitting one single FASTA-formatted DNA sequence per analysis, either by directly pasting the sequence into the input text box or by uploading a plain text file containing the FASTA sequence via the upload button. Users may optionally provide an email address to receive prediction results automatically; otherwise, they can retain the page open and view results directly upon completion. After clicking the Submit button, the platform executes a standardized bioinformatics pipeline, including gene prediction with Prodigal, functional annotation with HMMER, feature encoding, and model inference using the pre-trained XGBoost model. The output includes key analytical logs such as feature count, model loading status, and the primary result: the predicted optimal growth pH corresponding to the input genome. The platform is freely accessible, optimized for both desktop and mobile devices, and maintains stable server performance with uptime > 99%.

## Discussion

### Advantages of BactoGenopH: a pioneering tool for bacterial optimal pH prediction

BactoGenopH fills a critical gap in microbial biotechnology as the first dedicated tool for predicting bacterial optimal growth pH from genomic data, addressing key limitations of prior theoretical research [19] that lacked translational utility: (1) Grounding in measured phenotypes: Unlike prior theoretical studies that relied on indirect pH proxies (e.g., environmental abundance, phylogenetic inference), our dataset integrates only directly measured optimal growth pH values paired with standardized GTDB genomes. This ensures predictions align with true bacterial growth traits rather than inferred associations. (2) Comprehensive genomic features: Prior theoretical work often focused on preselected core genes or limited taxonomic groups, restricting generalizability. In contrast, BactoGenopH retains genome-wide high-importance genes (5,485 genes with importance scores above the mean) identified via XGBoost feature selection, enhancing applicability across diverse bacterial lineages—including niche-specific taxa with unique pH adaptation mechanisms (e.g., acidophilic Lactobacillaceae, alkaliphilic Alkalibacterium). The model’s superior performance (test set MAE = 0.477, MSE = 0.443; validation set MAE = 0.492, MSE = 0.482) outperforms prior work (e.g., Ramoneda et al. [19] reported MAE = 0.63 for pH range prediction), underscoring the value of our curated dataset and XGBoost-based feature modeling. Notably, the high accuracy (88.82% test set, 89.37% validation set) within a ± 1 pH unit tolerance (Table 3) provides actionable precision for applied scenarios.

### Implications for synthetic biology

The core value of BactoGenopH lies in bridging genomic-phenotypic gaps to accelerate synthetic biology innovations. Below, we link our results to two high-impact applications, leveraging the dataset’s taxonomic relevance (e.g., Lactobacillaceae) and model-derived mechanistic insights:

#### Rational design of pH-adapted probiotics

Probiotic efficacy in livestock depends on tolerance to the acidic gastric environment and the dynamically changing intestinal pH gradient—ranging from slightly acidic to neutral-alkaline across the small to large intestine [22, 24]. Our results highlight BactoGenopH’s utility for probiotic development in three key ways: (1) targeted strain selection: Predicting optimal growth pH from genomic data enables prioritization of strains matching gut niches—for example, *Lactobacillus* strains with predicted acid tolerance for gastric survival; (2) engineered resistance: Key genes identified via SHAP analysis (e.g., MgtE) provide actionable targets; enhancing MgtE expression in *Lactobacillus* could improve Mg^2^^+^ uptake and acid tolerance, increasing survival in the gastric environment; and (3) unculturable microbe screening: The model’s ability to process MAGs unlocks novel probiotic candidates from gut metagenomes, a vast reservoir previously inaccessible via traditional culture methods.

#### Optimization of silage fermentation

Silage quality relies on acidophilic LAB that lower pH to inhibit spoilage, but suboptimal pH adaptation of LAB consortia can hinder microbial community dominance, causing incomplete fermentation and diminished silage nutritional and storage quality [38]. Our taxonomic and phenotypic analyses confirm the relevance of our dataset to silage microbiology (e.g., Lactobacillaceae (67) accounting for 1.93% of samples (3,476)). BactoGenopH supports silage optimization by: (1) precision consortia formulation: Predicting optimal growth pH of LAB isolates or silage metagenome-derived MAGs enables mixing strains with complementary pH ranges, avoiding excessive or insufficient acidification; (2) enhanced LAB engineering: Key acid tolerance genes (e.g., Nramp, KdpA) identified in our SHAP analysis can be overexpressed to improve acid production and persistence; and (3) functional composite inoculant design: Silage additives often include non-LAB taxa with specific benefits (e.g., *Bacillus*) alongside LAB. BactoGenopH enables rational design of such composites by predicting pH adaptation of all strains, ensuring compatibility with the acidic silage environment and meeting production needs.

### Limitations and future directions

While BactoGenopH represents a significant advance, our results highlight opportunities for improvement. First, extreme pH group sample size: The small number of extreme acidophiles (*n* = 16) and alkaliphiles (*n* = 13) limits model accuracy for these taxa. Future work will expand the dataset by curating additional experimental pH data from literature and databases (e.g., expanding MAG inclusion beyond two samples/more strains) to enhance coverage of extremophilic lineages. Second, environmental factor integration: Our current model focuses on genomic features, but pH-dependent growth is influenced by temperature, nutrient availability, and oxygen levels. Integrating these variables into future models will improve prediction accuracy in complex real-world environments (e.g., rumen, silage silos). Third, taxonomic expansion: Archaea (e.g., rumen methanogens) and fungi (e.g., yeast probiotics) play critical roles in biotechnology but are not included in our current bacterial-focused model. Extending BactoGenopH to these taxa will broaden its utility. Fourth, synthetic biology tool integration: Developing a module to link SHAP-identified key genes (e.g., Na_Ala_symp, MgtE) with CRISPR-Cas9 engineering tools will enable direct design of expression vectors, streamlining microbial engineering workflows.

## Conclusion

BactoGenopH is a user-friendly, high-accuracy web platform for predicting bacterial optimal growth pH from genomic data. By grounding predictions in measured phenotypes and identifying functionally relevant key genes, BactoGenopH bridges genomic-phenotypic gaps and provides a foundation for synthetic biology research. Its applications—ranging from probiotic design to silage fermentation optimization—align with the transformative potential of synthetic biology to address global agricultural challenges. As a free, open-access resource, BactoGenopH empowers researchers worldwide to accelerate trait-driven microbial research and contribute to sustainable agriculture.

## Supplementary Information

Additional file 1: Fig. S1. Distribution plot of all phyla by total count of representative genomes matched to optimal growth pH. Fig. S2. Distribution plot of the top 50 classes by count of representative genomes matched to optimal growth pH. Fig. S3. Distribution plot of the top 50 orders by count of representative genomes matched to optimal growth pH.

Additional file 2: Table S1. Distribution number of all phyla by total count of representative genomes matched to optimal growth pH. Table S2. Distribution number of all classes by total count of representative genomes matched to optimal growth pH. Table S3. Distribution number of all orders by total count of representative genomes matched to optimal growth pH. Table S4. Distribution number of all families by total count of representative genomes matched to optimal growth pH. Table S5. Distribution number of all genera by total count of representative genomes matched to optimal growth pH. Table S6. Gene set common to seven bacterial groups. Table S7. Gene set not present in acidophile. Table S8. Gene set not present in alkaliphile. Table S9. Gene set unique to neutralophile.

## Data Availability

All bacterial genomic information and optimal growth pH data associated with this study are publicly available at our developed database website (http://silagedb.com/Optimal_pH/). The original data tables and associated files used for model training in this study are available for download from Figshare (https://doi.org/10.6084/m9.figshare.30937958) and GitHub (https://github.com/ChenHuilong1223/BactoGenopH).

## Abbreviations

MAE: Mean absolute error
MAG: Metagenome-assembled genomes
MSE: Mean squared error
*R*^2^: Coefficient of determination
RMSE: Root mean squared error
SHAP: SHapley Additive exPlanations
