# Supplementary material for: Machine learning-based linking of bacterial genomes to optimal growth pH: a foundation for rational microbial engineering
Source: J Anim Sci Biotechnol. 2026 Jun 11;17:116. doi: 10.1186/s40104-026-01434-7 (PMC13255231; doi:10.1186/s40104-026-01434-7)
Supplement: Supplementary file 1 — Additional file 1: Fig. S1. Distribution plot of all phyla by total count of representative genomes matched to optimal growth pH. Fig. S2. Distribution plot of the top 50 classes by count of representative genomes matched to optimal growth pH. Fig. S3. Distribution plot of the top 50 orders by count of representative genomes matched to optimal growth pH. [file 40104_2026_1434_MOESM1_ESM.docx]

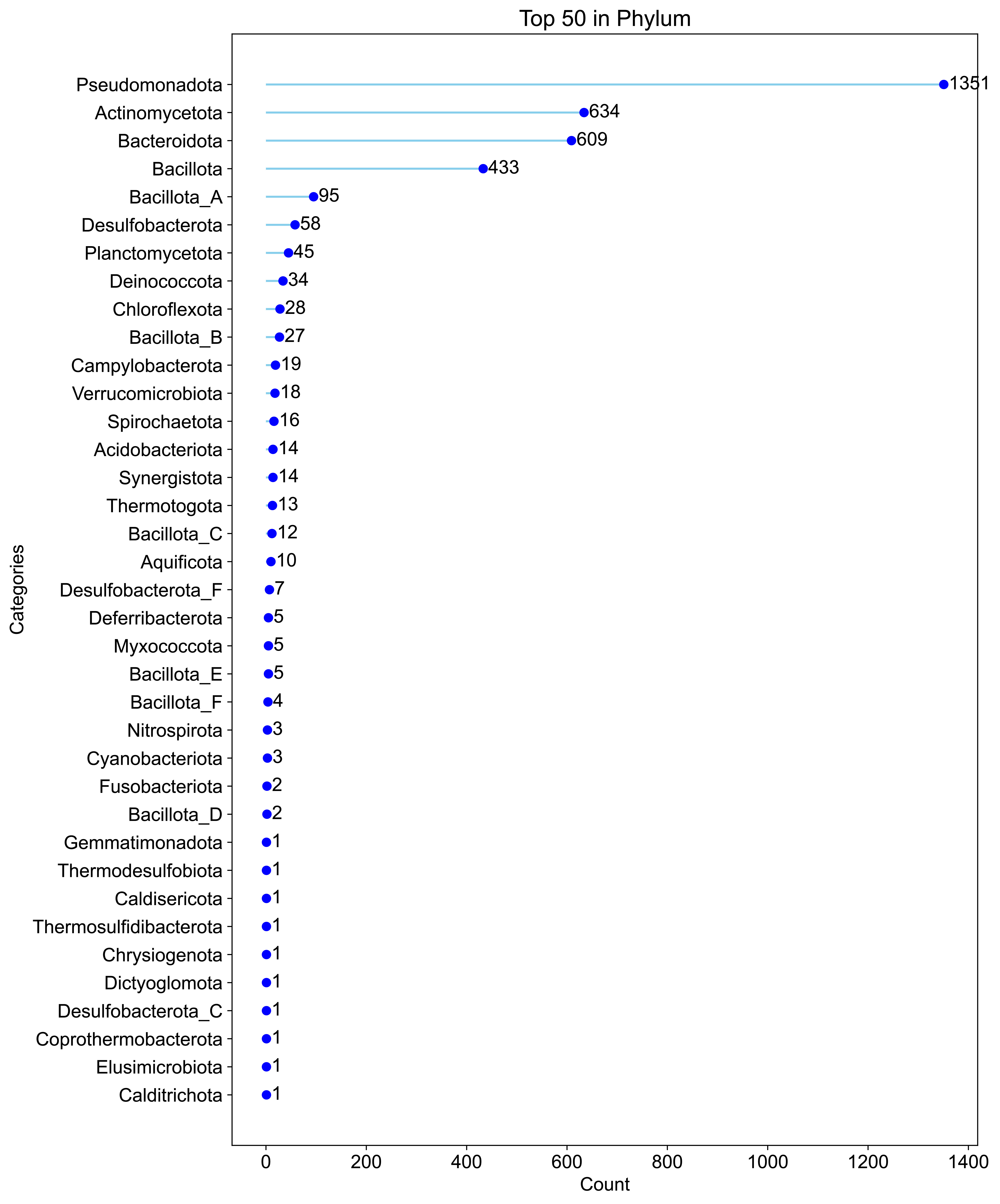


**Fig. S1** Distribution plot of all phyla by total count of representative genomes matched to optimal growth pH.


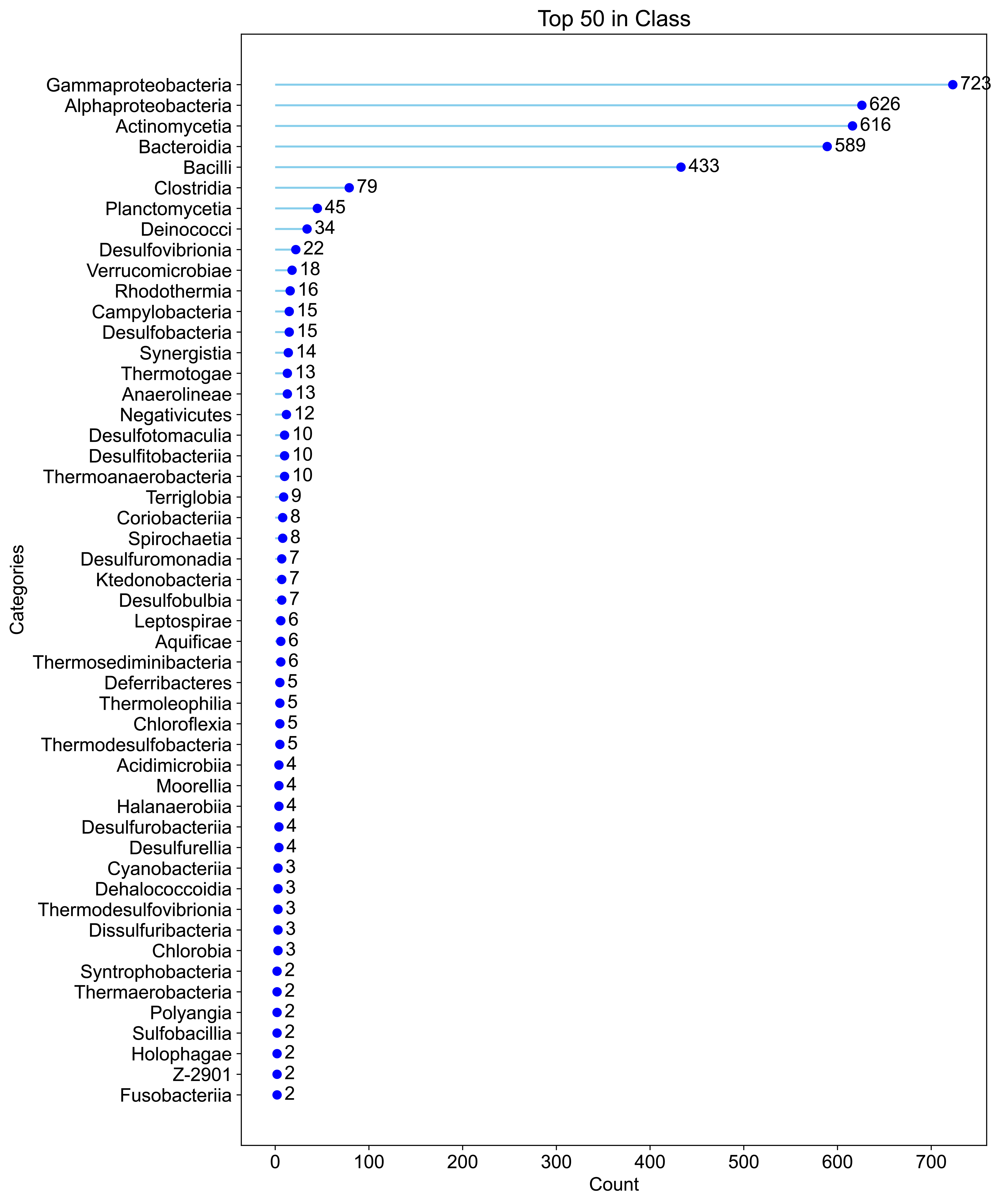


**Fig. S2** Distribution plot of the top 50 classes by count of representative genomes matched to optimal growth pH.


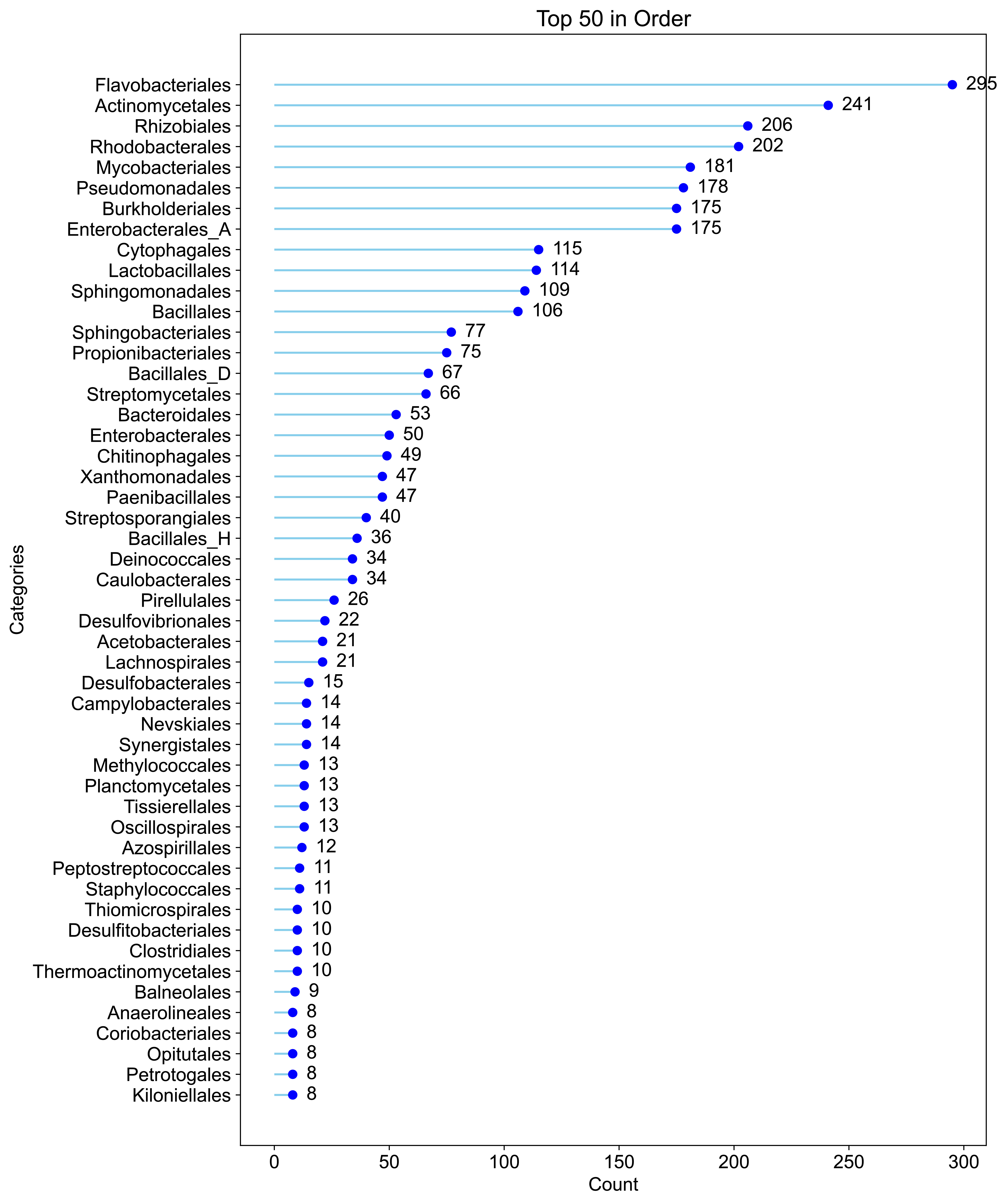


**Fig. S3** Distribution plot of the top 50 orders by count of representative genomes matched to optimal growth pH.
